# Management of Laryngotracheal Stenosis in Obesity. Is This Another Co-morbidity that Can Be Improved with Weight Loss Following Bariatric Surgery?

**DOI:** 10.1007/s11695-021-05647-9

**Published:** 2021-08-14

**Authors:** Matyas Fehervari, Shivali Patel, Rebecca Towning, Kevin Haire, Chadwan Al Yaghchi, Atika Sabharwal, Guri Sandhu, Evangelos Efthimiou

**Affiliations:** 1 Chelsea and Westminster Hospital, London, UK; 2 Imperial College London, London, UK; 3 National Centre for Airway Reconstruction, Imperial College Healthcare NHS Trust, London, UK

**Keywords:** Bariatric Surgery, Laryngotracheal stenosis, Airway stenosis, LTS, Comorbidity, Improved by bariatric surgery

## Abstract

**Purpose:**

Bariatric surgery improves several obesity-related comorbidities. Laryngotracheal stenosis is a rare condition that is usually managed with repeated endoscopic airway interventions and reconstructive airway surgery. The outcome of these definitive operations is worse in individuals with obesity. There are no studies investigating the effect of weight loss following bariatric surgery in the management of laryngotracheal stenosis.

**Materials and Methods:**

In an observational study, consecutive patients with a BMI over 35 kg/m^2^ and laryngotracheal stenosis were prospectively recruited to a bariatric and airway stenosis database in two tertiary care centres. Patients were treated with laparoscopic Roux-en-Y gastric bypass or sleeve gastrectomy and control subjects were managed conservatively.

**Results:**

A total of eleven patients with an initial body mass index of 43 kg/m^2^ (37–45) were enrolled to this study. Six patients underwent bariatric surgery and five subjects were treated conservatively. After 12 months, the total weight loss of patients undergoing bariatric surgery was 19.4% (14–24%) whilst 2.3% (1–3%) in the control group. The annual number of endoscopic airway interventions following bariatric surgery reduced (*p* = 0.002). Higher weight loss in patients led to less frequent interventions compared to control subjects (*p* = 0.004). Patients undergoing laryngotracheal reconstruction following bariatric surgery needed less endoscopic intervention, an annual average of 1.9 interventions before vs 0.5 intervention after. Conservatively managed control subjects required more frequent endoscopic intervention, 1.8 before vs 3.4 after airway reconstruction.

**Conclusion:**

Bariatric surgery reduced the number of endoscopic airway interventions and enabled patients to undergo successful definitive airway reconstructive surgery.

## Introduction

Obesity is associated with multiple comorbidities such as diabetes, obstructive sleep apnoea, cancer, and cardiovascular disease [1]. These conditions are commonly deteriorating with worsening of obesity whilst weight loss leads to remission of comorbidities [2, 3]. Laryngotracheal stenosis (LTS) is a rare but often severe condition described as narrowing of the laryngo-tracheobronchial tree. Historically intrinsic LTS is associated with infectious disease such as tuberculosis; however, currently most cases are secondary to prolonged intubation, tracheostomy, or following burn injury [4, 5].

The most effective treatment of morbid obesity is bariatric surgery (BS) which results in long-term and sustainable weight loss [6, 7]. Obesity-related all-cause mortality and morbidity shows a decrease following BS [8]. Most guidelines such as NICE or NIH recommends metabolic surgery for patients with BMI over 40 kg/m^2^ or BMI greater than 35 kg/m^2^ in patients with weight-related comorbidities [9, 10].

There is an increase prevalence of obesity in individuals with LTS. Increased neck circumference secondary to obesity is associated with higher number of cartilage fractures, impaired healing, and worsened outcomes following airway reconstruction [11–13]. Reconstructive surgery in those with co-existing severe obesity may not be feasible at all secondary to high anaesthetic risks and poor surgical outcomes; hence, it is not routinely performed [12–15]. Physiologically obese individuals have reduced total lung capacity, functional residual capacity, vital capacity, and elevated intrathoracic pressure causing central airway narrowing [16, 17]. Individuals with obesity and LTS have significant respiratory and airway symptoms leading to decrease in physical capability and worsening of obesity and LTS [18].

Historically, some airway diseases, for example obstructive sleep apnoea (OSA), were treated with airway reconstruction in obese individuals [19]. This was described as a successful treatment at the time. However, with the introduction of metabolic surgery, airway reconstruction is not performed for OSA in these patients any more as bariatric surgery is more effective at resolving OSA [20, 21].

Individuals with obesity and LTS have been treated either conservatively with regular endoscopic interventions or in some cases with reconstructive surgery, accepting a higher operative risk and poorer outcome. The effect of metabolic surgery on the outcome of airway reconstruction and endoscopic intervention in obese individuals has not been evaluated yet. In an observational study, the frequency of endoscopic interventions was recorded in morbidly obese individuals with LTS before and after BS and conservative weight loss interventions.

## Materials and Methods

A review of a prospectively collected bariatric and airway stenosis database was carried out across two tertiary centres screening for consecutive individuals with airway stenosis considered for weight loss surgery between 2014 and 2020. One of the centres is a national referral centre for airway stenosis patients and the other unit a tertiary centre for bariatric surgery. All patients were discussed in the high-risk bariatric multidisciplinary team meeting with ENT input and underwent comprehensive preoperative assessment. Patients with body mass index (BMI) higher than 35 kg/m^2^ were considered for bariatric intervention but only recommended surgery by MDT if they fulfilled criteria indicated in the current NICE guidelines [10]. If the individual had a BMI between 35 and 40 kg/m^2^, but did not have any medical condition that can be evidently improved with BS, life time modification was recommended in form of dietary consultation and follow-up by dieticians specialized in obesity. As both obesity and airway stenosis represent a significant surgical and anaesthetic challenge, patients eligible for surgery were further assessed on a Consultant-led anaesthetic clinic [12, 22].

Perioperative anaesthetic data, medical co-morbidities, detailed ENT data including the frequency of endoscopic interventions, type of bariatric surgery along with weight loss, and any adverse events were recorded. Endoscopic interventions were steroid injection, laser radial incisions, and balloon dilation. In patients with significant perioperative risks, an airway optimization intervention was carried out by the ENT team prior to bariatric surgery. Histamine releasing drugs were avoided and anaesthesia was maintained with sevoflurane to minimize any airway irritation.

Weight loss was assessed as previously described by Brethauer et al. [23]. Percentage of total weight loss (TWL%), percentage excess weight loss (EWL%)**,** and BMI were calculated at 3, 6, and 12 months after surgery. For individuals not having surgery**,** initial weight was registered and recorded a year later. Patients undergoing bariatric surgery were followed up in outpatient clinic by registering their weight loss prospectively into our database. Airway interventions were also registered prior to referral for BS. Patients were discharged back to ENT 12**–**18 months following definitive **b**ariatric **s**urgery and primarily followed up by the ENT team.

### Surgical and Anaesthetic Care

All patients were initially enrolled to the bariatric pathway before considering definitive airway reconstruction. For individuals with less severe airway stenosis and well controlled, moderate co-morbidities laparoscopic Roux-en-Y gastric bypass (LRYGB) was offered. Patients with more significant stenosis, poorly controlled or severe comorbidities, laparoscopic sleeve gastrectomy (LSG) was performed in the first instance. Depending on postoperative weight loss and improvement to comorbidities, a conversion to LRYGB was offered at a later stage. Once sufficient weight loss is achieved (30–40%EWL), symptomatic patients were offered airway reconstruction. Active smokers were excluded from bariatric surgical intervention.

### Statistical Analysis

Statistical analysis was performed with Microsoft Office for Mac (2019) and SPSS for Mac OSX 25.0.0 (SPSS Inc., Chicago, IL) software products. As the number of variables was low, percentages are avoided. For the demonstration of the effect of BS on LTS, the frequency of dilatation over 12 months was calculated by dividing the number of dilatations with the length of the investigated period in years. We used non-parametric Mann–Whitney’s U test to compare two independent groups. Statistical analyses were performed using two-tailed tests and *p* < 0.05 was considered significant. Values presented in the text are median and interquartile range in brackets unless otherwise stated. The study protocol was approved by Research and Development Office at Chelsea and Westminster Hospital (Reference number: PCD906).

## Results

There were 11 patients with LTS considered for weight loss intervention, 10 females and 1 male during the study period. The median length of the study was 64.1 (42–88) months, and the median follow-up after weight loss intervention was 49.1 (16–56) months. Six patients underwent bariatric surgery, 3 patients did not fulfil the NICE guidelines for bariatric surgery, and 2 patients decline to have a weight loss procedure. None of our patients suffered from OSA or Asthma. Out of the 6 patients, 2 had tracheal stenosis and 4 had subglottic stenosis. The causes of the stenosis were prolonged ventilation in 2 cases, autoimmune in 2 cases, and idiopathic in further 2 cases. The patient’s clinical characteristics are displayed in Table 1. None of the 6 patients undergoing surgery was considered suitable for definitive airway reconstruction prior to weight loss procedure and 2 of the subjects with more severe LTS and other significant comorbidities such as super obesity and cardiovascular disease were considered not fit for LRYGB despite this would have been the preferred surgical option. An endoscopic airway optimization was carried out in these individuals before undergoing a sleeve gastrectomy. A single patient started on conservative weight loss management lost to follow-up and not included in the study.

**Table 1:** Patient characteristics. Number of individuals suffering from common obesity related comorbidities in patients and controls

	Patients (underwent BS)	Controls (no BS)
Patients	6	5
Female	6	4
Diabetic	2	1
Smoker at presentation	0	1
Hypertension	2	1
Dyslipidaemia	3	2
GORD	2	3
Depression	2	0
NAFLD	1	0

No advanced airway management was required in the perioperative phase for any of the patients. All patients were recovered on the high dependency unit without suffering any postoperative airway or surgical complications.

### Weight Loss

The initial BMI across all groups was 43 kg/m^2^, 43.9 (42–50) kg/m^2^for patients, and 37.2 (35–46) kg/m^2^ for controls. Individuals undergoing non-surgical weight intervention had weights recorded at the time of bariatric referral and 1 year later. Their median BMI was 35.9 (34–45) kg/m^2^ after 12 months which corresponds to a 2.3 (1–3) TWL% and 7.9 (2–10) EWL%. The weight loss data for patients undergoing bariatric surgery is displayed in Table 2. One of the two individuals undergoing LSG was converted to LRYGB after 12 months and the other one after 24 months. Patient converted after 12 months achieved further 24.5 TWL% and 50.3 EWL%, whilst patient converted after 24 months achieved 14.5 TWL% and 40.6 EWL% with LRYGB.

**Table 2:** Weight loss following bariatric surgery in patients with LTS at different time points. Values are median and interquartile range

	Initial	3 m	6 m	12 m
BMI (kg/m^2^)	43.9 (42–50)	38.3(37–43)	36.7 (33–41)	36.3 (32–41)
EBMIL	N/A	30.3 (26–34)	40.6 (32–49)	42.5 (32–55)
TWL%	N/A	12.7 (12–14)	16.8 (15–21)	19.7 (14–24)
EWL%	N/A	30.3 (26–34)	40.6 (32–49)	42.5 (32–55)

### Airway Interventions

**Fig. 1:**
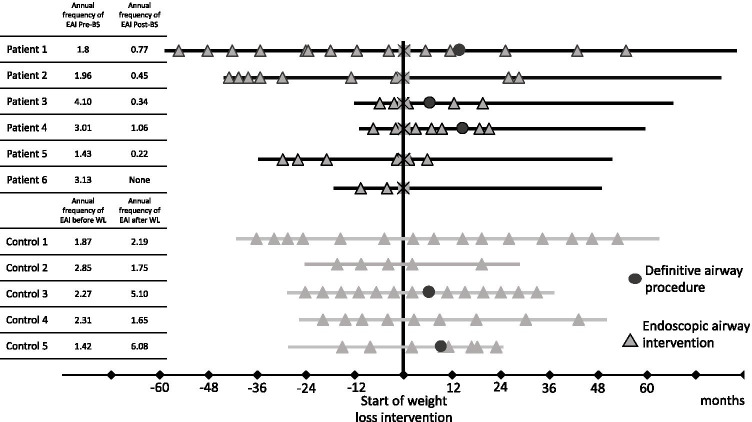
Annual frequency of endoscopic interventions in patients and controls. Each line represents and individual patient. *EAI, endoscopic airway intervention

The number of endoscopic airway interventions and definitive reconstructions was registered during the period of the study. Annual number of endoscopic airway interventions were recorded for each patient and displayed on Fig. 1.

In patients undergone bariatric surgery, a significant difference was found in the frequency of endoscopic airway procedures before and after BS (*p* = 0.002). Comparing the frequency of airway interventions between controls and patients prior to BS suggested no difference (*p* = 0.429). However, comparing the frequency of dilatation of patients undergone bariatric surgery to patients treated conservatively, the frequency of endoscopic procedures was significantly lower following weight loss surgery (*p* = 0.004). Comparing the frequency of endoscopic airway interventions prior to BS to the entire study period of controls (only conservative weight interventions), there were no significant difference (*p* = 0.792).

In subjects undergoing BS, three patients proceeded to have airway reconstruction and three did not require regular endoscopic interventions and not qualified for definitive airway reconstruction anymore***.*** The timing of airway reconstruction was 6 months in one case and 14 months in two cases after BS. The three patients undergoing reconstruction on average needed less frequent endoscopy than before, respectively, 1.9 and 0.5 dilatation per year. In control subject, airway reconstruction was attempted in 2 cases; however, this did not lead to decrease in the frequency of endoscopic airway procedures. In fact, on average these individuals required more frequent dilatation per year after airway reconstruction, respectively, 1.8 and 3.4.

## Discussion

This study looked at the potential benefit of weight-loss surgery in individuals with obesity and symptomatic LTS. Simultaneous presentation of these two conditions is not common which is reflected in the number of the cases identified in National Tertiary Centers over 6 years. However as described, individuals with obesity and LTS are increasingly affected by both conditions [18]. As this is the first article describing the effect of BS on narrowing of the airway, it is currently not considered obesity-related co-morbidity. Patients currently presenting with BMI between 35 and 40 kg/m^2^ with no other comorbidities are not fulfilling the NICE guidelines to qualify for bariatric surgery. Three of eleven subjects were in this category. Some patients in this study were not ready to accept bariatric surgery without evidence demonstrating improvement of LTS. The general medical characteristics are typical for obese individuals with the exception of OSA. This is an important distinction in this group as overwhelming evidence suggests BS and weight loss as one of the most important treatments of this condition in individuals with obesity [24]. Interestingly, this was not always the case. The ear, nose, and throat specialists were treating OSA with definitive airway reconstruction prior to the wide spread of BS. Studies from those times suggested that reconstruction was a feasible option but did carry a risk of morbidity and mortality [19].

High-risk patients with significant comorbidities were initially treated with laparoscopic sleeve gastrectomy and preoperative endoscopic airway intervention to minimize the risk of the procedure. This strategy was successful as neither of these patients suffered higher than grade 2 complications according to the Clavien-Dindo classification [25]. Lifestyle modification and conservative weight loss measures were less effective in LTS patients than recorded previously for adults with morbid obesity only [26]. Similar findings were observed in subjects undergoing bariatric surgery with TWL% and EWL% being lower than what our institution has reported in patients without LTS (TWL% 32.7 ± 0.58, EWL% 70.4 ± 1.35%) [27]. Subjects initially undergoing a sleeve gastrectomy followed by conversion to LRYGB were followed up for the longest period of time and achieved the greatest weigh loss in our cohort.

Subjects undergoing BS needed significantly less frequent interventions after the surgery than before and significantly less frequent interventions after surgery than conservatively managed control patients. This demonstrates a clear improvement of LTS following BS. Some patients improved to a level that they did not require further interventions to manage their airway symptoms whilst others underwent definitive reconstruction. These reconstructions resulted in less frequent endoscopic dilatations compared to control subjects where reconstructions actually led to more frequent procedures. This suggests that BS improves the outcome of definitive airway reconstruction in obese individuals with LTS and confirms previous findings suggesting poor outcome without weight loss [15].

The limitation of our study is the small number of individuals enrolled. However, these conditions are rare. Previous studies discussing LTS alone usually report on a similar number of cases. Improvements of quality of life and long-term follow-up are important factors that will also need to be established in the future.

In summary, this is the first study investigating the effect of bariatric surgery on laryngotracheal stenosis and comparing outcomes to control population. Bariatric surgery reduces the number of endoscopic airway interventions and enables patients to undergo successful definitive airway reconstructive surgery with lesser need for dilations following surgical airway reconstruction. These findings suggest that obese individuals with BMI over 35 kg/m^2^ with LTS should be considered for bariatric surgery. We recommend that these patients are referred to a bariatric surgeon early on, even if they are not considered suitable for definitive airway reconstruction. The type of BS should be based on fitness for surgery and surgeon’s individual experience. Laparoscopic Roux-en-Y gastric bypass is an appropriate choice in most cases. Laparoscopic sleeve gastrectomy is an alternative option for more comorbid patients. Airway optimization prior to bariatric surgery is required to ensure an adequate and stable airway.
